# The diagnosis of hepatic fibrosis by magnetic resonance and near-infrared imaging using dual-modality nanoparticles†

**DOI:** 10.1039/c7ra10847h

**Published:** 2018-02-12

**Authors:** Yunfang Li, Wenting Shang, Xiaoyuan Liang, Chaoting Zeng, Mingming Liu, Sudan Wang, Hongjun Li, Jie Tian

**Affiliations:** Department of Radiology, Beijing YouAn Hospital, Capital Medical University 8 Xitoutiao, Youanmen, Fengtai District Beijing China 100069 lihongjun64@126.com +86 10 63051128 +86 10 83997337; Key Laboratory of Molecular Imaging, Institute of Automation, Chinese Academy of Sciences Zhongguancun East Road #95, Haidian Dist Beijing 100190 P. R. China jie.tian@ia.ac.cn +86 10 62527995 +86 10 82618465

## Abstract

Hepatic fibrosis (HF), as the only reversible process of chronic liver disease, remains a big diagnostic challenge. Development of noninvasive and effective methods to assess quantitatively early-stage HF is of great clinical importance. Compared with conventional diagnostic methods, near-infrared fluorescence imaging (NIR) and magnetic resonance imaging (MRI) could offer highly sensitive and spatial resolution signals for HF detection. However, precise detection using contrast agents is not possible. Superparamagnetic iron oxide (SPIO) nanoparticles have low toxicity, high sensitivity and excellent biocompatibility. Integration of Fe_3_O_4_ nanoparticles and indocyanine green (ICG), coupled with targeting ligand of integrin α_v_β_3_, arginine–glycine–aspartic acid (RGD) expressed on hepatic stellate cells (HSCs), were used to detect HF. Both *in vivo* and *in vitro* results showed that the SPIO@SiO_2_–ICG–RGD had high stability and low cytotoxicity. The biodistribution of SPIO@SiO_2_–ICG–RGD was significantly different between mice with HF and healthy controls. SPIO@SiO_2_–ICG–RGD was characterized and the results of imaging *in vitro* and *in vivo* demonstrated the expression of integrin α_v_β_3_ on activated HSCs. These data suggest that our SPIO@SiO_2_–ICG–RGD probe could be used for the diagnosis of early-stage HF. This new nanoprobe with a dual-modality imaging approach holds great potential for the diagnosis and classification of HF.

## Abstract 2

Schematic diagram for the synthesis of SPIO@SiO_2_–ICG–RGD.

## Graphical abstract

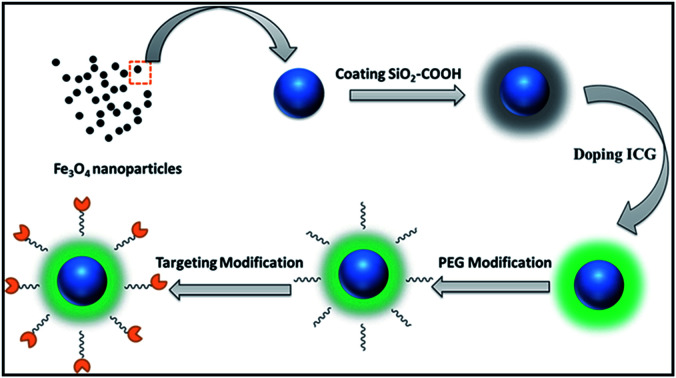

## 1. Introduction

**Scheme 1:**
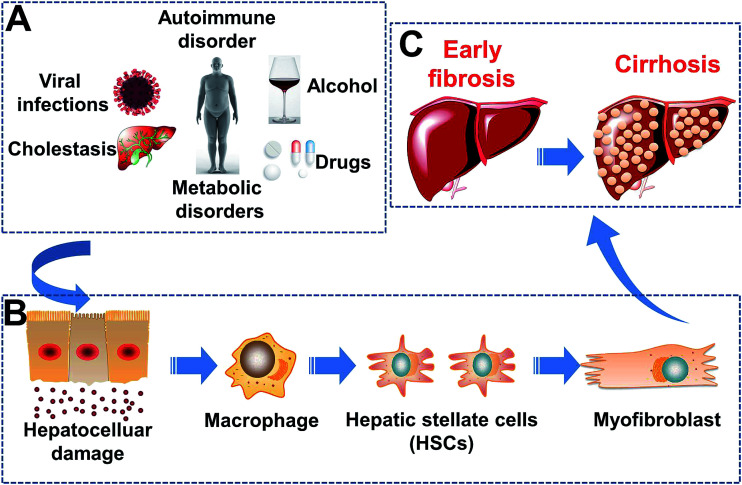
Pathogenesis of hepatic fibrosis. (A) Etiology of liver damage. (B) Liver damage at the cellular level. (C) Development of hepatic fibrosis.

Hepatic fibrosis (HF) is a key stage in the progression of chronic liver disease,^1,2^ which can aggravate to cirrhosis and liver cancer. As shown in Scheme 1, liver injury can be initiated by several precipitating factors. Liver injury initiates a cascade of fibrogenic processes initiated by inflammatory and fibrogenic signals.^3,4^ In response to a persistent liver injury, macrophages^5,6^ initiate the recruitment and transformation of resident quiescent hepatic stellate cells (HSCs)^7,8^ to a myofibroblast phenotype, which is highly activated, proliferative, motile, and contractile.^9^ The cells of this phenotype are a major source of a redundant extracellular matrix (ECM).^10,11^ Accumulation of the ECM is the main reason for HF induction.^3^ In this process, integrin α_v_β_3_ is expressed by HSCs. Integrin α_v_β_3_ enhances the adhesion and migration of HSCs, which bind to the ECM,^12–14^ whereas integrin α_v_β_3_ is not expressed by hepatocytes. Hence, integrin α_v_β_3_ can be used as a unique target to activate HSCs, which induce fibrogenesis in the liver. The targeting ligand of integrin α_v_β_3_ is a sequence of three amino acids: arginine–glycine–aspartic acid (RGD).^15–17^

Recent studies have reported that HF is reversible and treatable at an early stage.^18–20^ Therefore, early detection of HF in patients with chronic liver diseases is important for clinical management and treatment decisions. Liver biopsy is used as the “gold standard” to assess HF stage. However, it is an invasive procedure with some significant complications, so it is not usually accepted by patients.^21^ Laboratory examinations and imaging such as ultrasound (US), US shear-wave elastography, computed tomography, and magnetic resonance imaging (MRI) are often used in the diagnosis of liver cirrhosis and related complications. However, these methods have drawbacks regarding early diagnosis. Hence, for most patients, lesions are not identified at an early stage.^22–25^

The development of multifunctional probes can combine the advantages of two or more diverse imaging modalities to make imaging more effective.^26,27^ Therefore, a multifunctional probe can be developed to target and image HF. This strategy could provide a novel method for HF detection to overcome the shortcomings of single-imaging modalities.

We hypothesized that a probe could combine two signals (MRI and near infrared fluorescence (NIR) imaging) to aid the diagnosis of HF.^28,29^ Superparamagnetic iron oxide (SPIO) nanoparticles have low toxicity, high sensitivity and excellent biocompatibility, and are the first T2 contrast agents for MRI to be used clinically.^30^ Compared with fluorescent quantum dots and cyanine dye, indocyanine green (ICG) is the only NIR organic dye approved by the US Food and Drug Administration (FDA) for medical imaging and diagnosis in clinical practice for more than 50 years.^31^ ICG and SPIO have disadvantages: a tendency to aggregate and degrade rapidly in aqueous solution; a tendency to clear rapidly from the body with a short half-life of 2–4 min; absence of active targeting; being prone to photo-bleaching. Hence, the most effective method is to establish novel particles combined with ICG and SPIO. Also, “hydrothermal synthesis” refers to the chemical reactions of substances in a sealed heated solution above ambient temperature and pressure.^32^

In this context, to improve the targeting ability of particles, an optimal target site should be selected for HF. As mentioned above, RGD can be used to target activated HSCs by binding to integrin α_v_β_3_ receptors because the integrin α_v_β_3_ is expressed on activated HSCs^33^ (the target cells in HF). Hence, we enabled the binding of RGD to the surface of nanoparticles.

Here, we report the synthesis of small-scale core–shell nanoparticles. These were engineered by conjugation with ICG and SPIO, which target integrin α_v_β_3_ due to its RGD sequence. Then, we evaluated the potential utility of the nanoprobe, SPIO@SiO_2_–ICG–RGD, using MRI/NIR for precise imaging guidance of early-stage HF in a mouse model. We found that the nanoparticles simultaneously possessed MRI/fluorescence imaging features in HF *in vivo*. Interestingly, a synergistic effect of the nanoparticles suggested that they have good biocompatibility and could realize imaging-guided location of HF.

## 2. Experimental

### 2.1. Materials and instrument

Tetraethylorthosilicate (TEOS), (3-aminopropyl)triethoxysilane (APTES) and pyrrole were obtained from Sigma-Aldrich (Saint Louis, MO, USA). 2-Methoxy (polyethyleneoxy) propyl trimethoxysilane (PEG-silane, 596–725 Da) and cetyltrimethylammonium bromide (CTAB, 98%) were purchased from Alfa Aesar China (Beijing, China). The RGD peptide (Arg–Gly–Asp, 5 mg, 98%) and Fe_3_O_4_–COOH (1 mg mL^−1^, Fe) nanoparticles were obtained from Thermo Scientific (Waltham, MA, USA). ICG solution (10 mg mL^−1^) was purchased from Dandong Medical and Pharmaceutical Co. Ltd. (Dandong, China). All other chemicals were of analytical grade and used without further purification. Deionized water (DI water) was obtained from a water-purification system (Millipore, Bedford, MA) with a resistivity of 18.2 MΩ cm^−2^.

Transmission electron microscopy (TEM) using a 1011 system (JEOL, Tokyo, Japan) and scanning electron microscopy (SEM) employing a S-4800 instrument (Hitachi, Tokyo, Japan) using an acceleration voltage of 100 kV enabled evaluation of the morphology and size of nanoparticles. The hydrodynamic particle size distribution was measured by dynamic light scattering (DLS) using a Zeta potential meter (ZEN 3600-nanoZS; Malvern Instruments, Malvern, UK). X-ray photoelectron spectroscopy (XPS) was done on an ESCALab™ 250Xi instrument (Thermo Scientific) using 200 W monochromatic Al Kα radiation. The ICG concentration was determined using an absorbance assay at 785 nm for ICG. UV-vis-NIR spectra were obtained with a Lambda 25 spectrophotometer (PerkinElmer, Waltham, MA, USA). Fluorescence images were obtained using an animal *in vivo* optical imaging system (IVIS Spectrum; PerkinElmer). MRI was done using an animal MRI scanner (Aspect, Shoham, Israel).

### 2.2. Synthesis of SPIO@SiO_2_–ICG–RGD

**Fig. 1:**
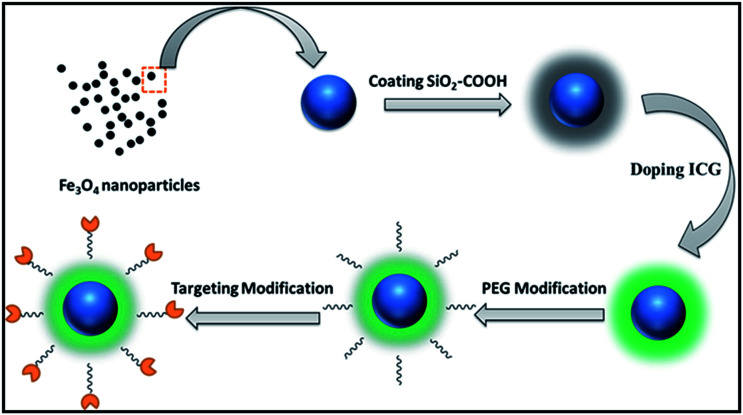
Synthesis of SPIO@SiO_2_–ICG–RGD (schematic).

The nanoprobe was synthesized following the scheme shown in Fig. 1. SPIO cores (Fe_3_O_4_ nanoparticles) were synthesized as described previously.^34^ After modification of Fe_3_O_4_ nanoparticles (which had a hydrodynamic diameter of 24 ± 4 nm), SiO_2_ nanocores were prepared using a conventional method. Briefly, the reactive solvents were prepared by adding ethanol (3 mL) to DI water (2 mL). After stirring for 0.25 h by magnetic stirrers at room temperature, 5 mL of Fe_3_O_4_–COOH (1 mg mL^−1^, Fe_3_O_4_) was added to the solvent, followed by 0.3 mL of ammonia solution (25%). About 30 μL of TEOS was added dropwise and allowed to react followed by continuous stirring at 800 rpm for 3 h at 40 °C. An external magnetic field was used to collect solid products, and then rinsed thrice with DI water and ethanol. The precipitate was re-dispersed in 10 mL of ethanol.

Then, SPIO@SiO_2_ nanoparticles were coated with polyethylene glycol (PEG) MW-5000 using a dual-solvent exchange method. Approximately 0.01 mg mL^−1^ of ICG was added to 1 mL of the resulting solution (loading efficiency: 30%). The mixture was then placed on a shaker and incubated at room temperature overnight. The unloaded ICG molecules and SPIOs were removed by ultracentrifugation at 10 000 rpm. The RGD peptide was combined with SPIO@SiO_2_ nanoparticles using the NHS-EDS reaction and shaken for 6 h. The targeting probe was stored at room temperature within silver paper to protect it from light.

After the synthesis, for *in vivo* imaging, ≈100 μL of SPIO@SiO_2_–ICG–RGD (0.2 mg mL^−1^, Fe_3_O_4_) was used for each mouse, which contained 1 μg of ICG dye.

### 2.3. Cell-line culture, cytotoxicity assay and toxicity evaluation *in vivo*

A mouse HSC line, human HSC line (LX-2) and normal human liver cell line (HL7702) were purchased from China Infrastructure of Cell Line Resources (Beijing, China). Mouse HSC and HL7702 lines were cultured in H-Dulbecco's modified Eagle's medium (H-DMEM) (Gibco, Billings, MT, USA) supplemented with 10% fetal bovine serum (FBS) (Gibco) and 1% penicillin–streptomycin. LX-2 cells were cultured in L-DMEM (Gibco) supplemented with FBS (10%), basic fibroblast growth factor (bFGF), insulin, hydrocortisone, transferrin, streptomycin and penicillin. The cultured cells were maintained in 5% CO_2_ in a tissue culture incubator at 37 °C.

Mouse HSC cells, LX-2 cells and HL7702 cells were used to detect the toxicity of the SPIO@SiO_2_–ICG–RGD nanoprobe. The cytotoxicity assay of the nanoprobe was evaluated by the 3-(4,5-dimethylthiazol-2-yl)-2,5-diphenyltetrazolium bromide (MTT) assay (Thermo Scientific). Cells (mouse HSC, LX-2 or HL7702) were seeded in a 96-well tissue culture plate at 1 × 10^4^ cells per well using 200 μL of cell culture medium to disperse them as described previously.^17,18^ Before the experiment, the cells reached 60–80% confluence after incubation in 5% CO_2_ for 48 h (mouse HSC, LX-2) or 24 h (HL7702) 37 °C. After reaching 60–80%, the cells were incubated with SPIO@SiO_2_–ICG–RGD (10, 25, 50, 75, 150, and 300 μg mL^−1^, Fe_3_O_4_). After 24 h of incubation, 100 μL of fresh medium and 10 μL of MTT solution (5 mg mL^−1^ in phosphate-buffered saline (PBS)) was added to each well and cultured for an additional 4 h. After removal of the old medium, 120 μL of dimethyl sulfoxide was added to each well and the absorbance at 600 nm read using a microplate reader to calculate relative cell viabilities.

For evaluation of the toxicity of nanoparticles, an *in vivo* test was undertaken on 10 healthy mice separated randomly as a treatment group and control group (*n* = 5 per group). Approximately 200 μL (0.2 mg mL^−1^, Fe_3_O_4_) of SPIO@SiO_2_–ICG–RGD was delivered *via* the tail vein to the treatment group. After being raised conventionally for 4 weeks, blood was obtained from the hearts of mice and *in vivo* biochemistry tests undertaken.

### 2.4. *In vitro* cell fluorescence imaging

For fluorescence imaging *in vitro*, mouse HSC cells and LX-2 cells were incubated with SPIO@SiO_2_–ICG–RGD (50 μL; 0.2 mg mL^−1^, Fe_3_O_4_) for 2 h in a 96-well tissue culture plate. Before that, 1 × 10^5^ cells were cultured in 96-well microtiter plates for 24 h. Cells were washed thrice to stop uptake by the cell culture medium and twice with PBS. Then, 2 mL of the cell culture medium was added to the cell culture dish.

Then, a homemade stereoscopic fluorescence microscope was applied to observe cellular uptake of SPIO@SiO_2_–ICG–RGD at an excitation wavelength of 780 nm and emission wavelength of 840 nm.

### 2.5. Ethical statement of animal experiment and preparation of a mouse model

The experimental protocol was approved by the Institutional Animal Care and Ethics Committee of Capital Medical University (Beijing, China). Animal experiments were carried out in compliance with the relevant guidelines of the National Institutes of Health (Bethesda, MD, USA). The maintenance and care of animals ensured humane treatment, and all methods were carried out in accordance with relevant guidelines.

Five-week-old male Kunming mice were obtained from the Beijing Vital River Laboratory Animal Technology Co., Ltd (Beijing, China). The mouse model for HF was obtained from the Laboratory Animal Center of the Chinese Academy of Medical Sciences (Beijing, China). The mouse model was induced by intraperitoneal injection of carbon tetrachloride (CCl_4_). Approximately 2.5 μL of CCl_4_ (40%) per gram of body weight was intraperitoneally injected twice a week into mice (*n* = 20). The quality of life of mice was observed continuously until the HF model was established for the experiments. After ≈2 weeks, an early-stage HF mouse model was ready for the experiments, as confirmed by histology. Animal procedures were implemented under anesthesia (3% isoflurane–air mixture).

### 2.6. Fluorescence imaging of HF *in vivo*

Fluorescence imaging *in vivo* was undertaken on 10 mice with HF and 10 healthy mice (controls). Approximately 100 μL (0.2 mg mL^−1^, Fe_3_O_4_) of SPIO@SiO_2_–ICG–RGD was delivered *via* the tail vein. An IVIS Spectrum imaging system (PerkinElmer) was used to monitor the change in fluorescence signal in mice 0.5, 1, 3, 6, 12, 24, 48, 72, and 96 h post-injection with excitation and emission wavelengths of 790 nm and 828 nm, respectively. Imaging data were analyzed with IVIS Living Image v3.0 (PerkinElmer). The region of interest (ROI) was selected to measure the mean fluorescence intensity (MFI) of the liver. The target:background ratio (TBR) was obtained by dividing the MFI of the liver area by the corresponding body background area. To evaluate the targeting ability of RGD, imaging of the HF of mice treated with SPIO@SiO_2_–ICG *in vivo* was done.

### 2.7. MRI of HF *in vivo*

*In vivo* MRI data were obtained on a 1.0-T MRI system (Aspect). T2-weighted axial whole-liver MRI datasets were obtained using a fast spin echo-imaging sequence (TR/TE/TI = 1500 ms/20.7 ms/100 ms; flip angle = 90°; field of view = 40 × 40 mm; slice thickness = 1 mm; resolution = 1 × 1 mm; slices = 16). All were processed using the same scanning protocol. We monitored the change in the MRI signal in mice 1, 3, 6, 9, 12, 48, 72, and 96 h post-injection. The change in MRI contrast in the liver following injection of the targeting probe was calculated quantitatively using the ROI method with eFilm image processing software (Merge Healthcare Corp., Chicago, IL, USA). The mean MRI signal intensities of the ROI were calculated from the liver. Then, the normalized signal intensities (SIs) were obtained by comparing the SIs before and after that of the liver ROI. We used two methods to evaluate the SI. First, we used SNR (SI_liver_/SD_background_). Second, the values were calculated from the liver and corresponding muscle areas. The mean change in MRI contrast was calculated from five MRI slices of the entire liver and muscle with the same area of the ROI.

To evaluate the targeting ability of RGD, ≈100 μL of SPIO@SiO_2_–ICG was delivered by the tail vein to HF mice (*n* = 10). Then, *in vivo* imaging of HF was obtained from the IVIS Spectrum imaging system (PerkinElmer) and the 1.0-T MRI system (Aspect) using the method mentioned in Sections 2.6 and 2.7. The results of the experiment are given in ESI (Fig. S2 and S3†).

### 2.8. Probe biodistribution and histology

Mice were sacrificed following optical imaging/MRI. The heart, liver, spleen, lungs, and kidneys were harvested to determine the biodistribution of the probes and for histology. The optical images for assessing the fluorescence distribution were obtained using the IVIS Spectrum imaging system. Prussian blue staining was undertaken for the detection of iron in tissue sections. Tissue morphology was verified by hematoxylin and eosin (H&E) staining. Slices were examined using a digital microscope (QWin; Leica, Wetzlar, Germany).

### 2.9. Statistical analyses

Data are the mean ± standard deviation from at least three sample replicates. Statistical comparisons were conducted using Student's *t*-tests and paired *t*-tests with SPSS v17 (IBM, Armonk, NY, USA), and graphs were drawn using Prism 5 (GraphPad, La Jolla, CA, USA). *P* < 0.05 was considered significant.

## 3. Results

### 3.1. Synthesis and characterization of SPIO@SiO_2_–ICG–RGD

**Fig. 2:**
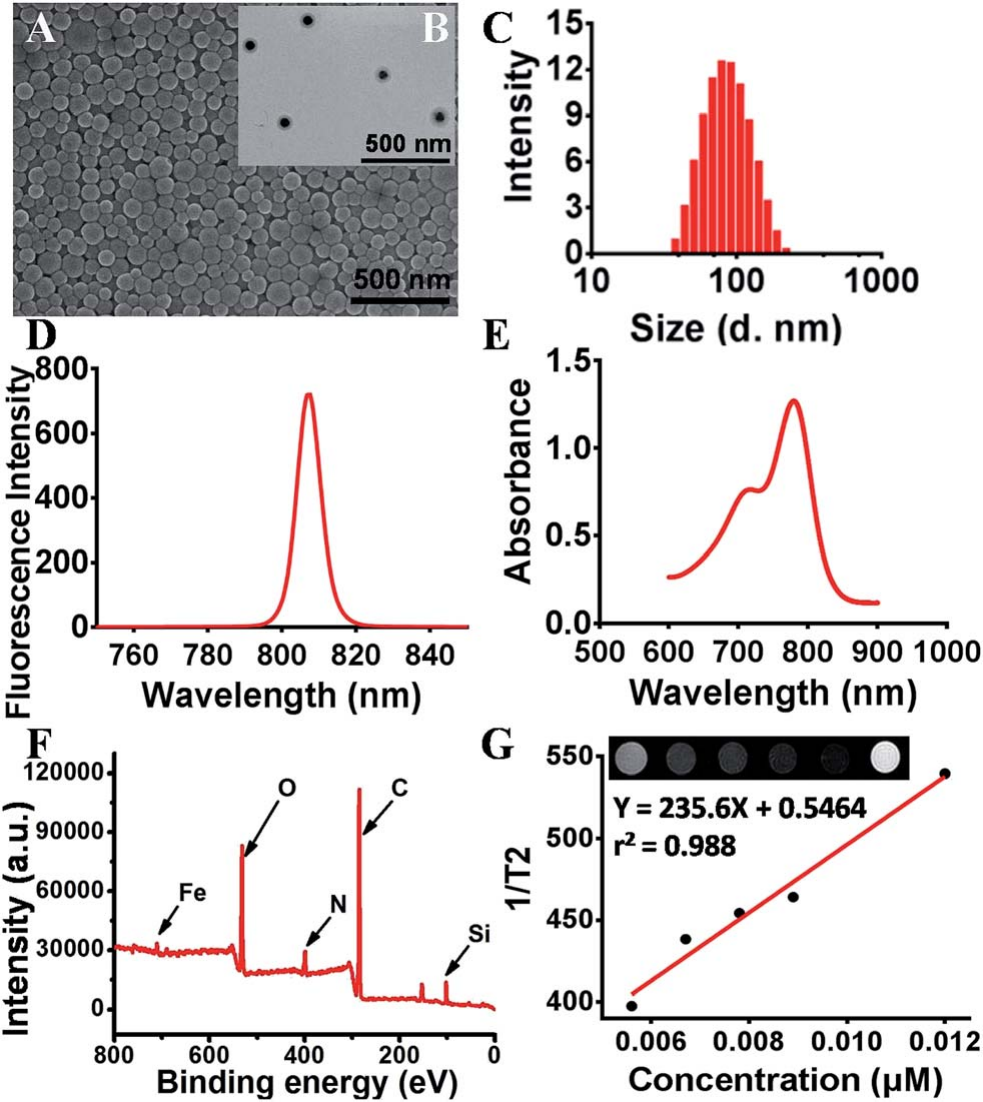
Characteristics of a dual-functional agent SPIO@SiO_2_–ICG–RGD. (A) SEM image of SPIO@SiO_2_–ICG–RGD. (B) TEM image of SPIO@SiO_2_–ICG–RGD. (C) DLS-measured diameters of SPIO@SiO_2_–ICG–RGD in PBS. (D) Spectra of SPIO@SiO_2_–ICG–RGD. (E) Visible absorption spectra of SPIO@SiO_2_–ICG–RGD. (F) XPS spectrum of SPIO@SiO_2_–ICG–RGD. (G) T2-weighted MRI and T2 relaxation for various concentrations of SPIO@SiO_2_–ICG–RGD.

The morphology of SPIO@SiO_2_–ICG–RGD was characterized using SEM (Fig. 2A) and TEM (Fig. 2B). SPIO@SiO_2_–ICG–RGD exhibited a typical spherical morphology with an average diameter of <100 nm (Fig. 2A and B). The average hydrodynamic diameter of SPIO@SiO_2_–ICG–RGD was −100 nm as measured by DLS (Fig. 2C). The excitation spectrum of SPIO@SiO_2_–ICG–RGD exhibited a fluorescence intensity at 807 nm (Fig. 2D). In the UV-vis spectrum, absorption peaks were detected at 712 nm and 779 nm, which corresponded to SPIO and ICG, respectively (Fig. 2E). XPS indicated that iron, carbon, oxygen, nitrogen, and silicon were present in the core–shell composite SPIO@SiO_2_ (Fig. 2F). Iron arose mainly from Fe_3_O_4_, which formed the core of the nanoprobe. Silicon came mainly from SiO_2_ nanosheets. Oxygen originated from the oxygen-containing functional groups in SiO_2_ and Fe_3_O_4_ nanoparticles. Carbon arose mainly from TEOS and alcohol, which provide carboxyl groups in the SiO_2_@Fe_3_O_4_ to be grafted next. Nitrogen may have originated from the residual ammonia solution. The XPS high-resolution spectra of Fe 2p, C 1s, Si 2p and O 1s from the fractured surfaces of SPIO@SiO_2_ are given in Fig. S1.† The Fe 2p spectrum (Fig. S1A†) for the composite consisted of two symmetrical broadened peaks with binding energies of 709.34 eV and 724.24 eV, which were attributed to Fe 2p_3/2_ and Fe 2p_1/2_, respectively. That was indicated for Fe^2+^ and Fe^3+^ in Fe_3_O_4_, and the mixed state of FeO and Fe_2_O_3_. These values agree very well with literature values.^35^ The C 1s spectrum of SPIO@SiO_2_ (Fig. S1B†) was separated into three peaks at binding energies of 284.27, 285.78, and 288.31 eV, which were attributed to SP^3^ ± C, C ± O and C=O groups, respectively. For the Si 2p and O 1s spectra in SPIO@SiO_2_, the strongest peaks were at 101.69 eV (Fig. S1C†) and 531.30 eV (Fig. S1D†), respectively, with the calculated content in nanoparticles being 17.82 wt% and 7.40 wt%, respectively. The relaxation times of the core Fe_3_O_4_ in the structure indicated that the paramagnetic characteristic would enhance the T2 contrast. The different MRI signals were captured using a 1.0-T MRI scanner (Fig. 2G). The variables exhibited a linear relationship, with *r*^2^ = 0.988 and *R*^2^ = 235.6 × 10^−3^ m s^−1^.

### 3.2. Toxicity to HSCs *in vitro* and *in vivo*

**Fig. 3:**
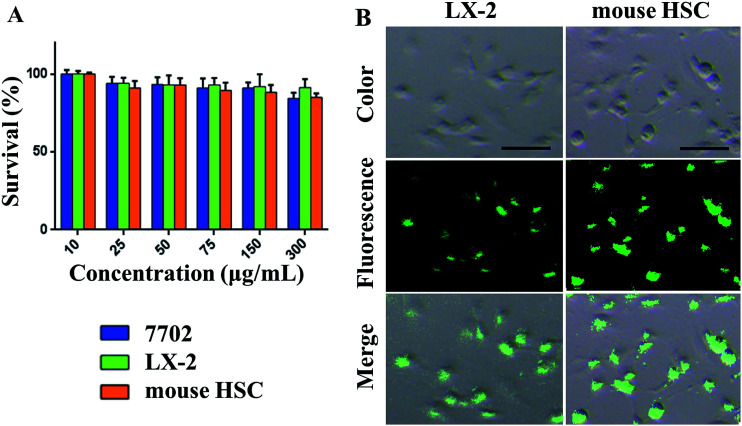
*In vitro* cytotoxicity and endocytosis evaluation of nanoparticles in cells. (A) Cell-viability assays (mouse HSCs, LX-2 cells and HL7702 cells) of SPIO@SiO_2_–ICG–RGD over various concentrations. (B) NIR images of mouse HSCs and LX-2 cells after incubation with SPIO@SiO_2_–ICG–RGD. Scale bar = 100 μm.

We investigated the safety of our nanomaterials, which is of utmost importance in biomedical applications. The toxicity of SPIO@SiO_2_–ICG–RGD was assessed using a microplate reader to ensure their safety and compatibility *in vivo*. The effects on HSC survival are shown in Fig. 3A. Survival of the three cell lines was >85%. Different concentrations resulted in a slight decrease in the C50 value from 10 to 300 μg mL^−1^. The cytotoxicity testing of SPIO@SiO_2_–ICG–RGD indicated that it had good biocompatibility for use *in vivo*.

Testing of SPIO@SiO_2_–ICG–RGD uptake revealed significantly high fluorescence intensity after 2 h of incubation with SPIO@SiO_2_–ICG–RGD in mouse HSCs and LX-2 cells (Fig. 3B). The RGD sequence in the probe could target both of these lines and bind specifically with the integrin α_v_β_3_ receptor, suggesting that the SPIO@SiO_2_–ICG–RGD probe had satisfactory specificity for HSCs (Fig. 3B). These results suggested that SPIO@SiO_2_–ICG–RGD could be used for NIR imaging or for observing HSCs in the liver of a living body.

The results of toxicity testing of SPIO@SiO_2_–ICG–RGD *in vivo* are shown in Table S1.† Apart from alanine aminotransferase (ALT), levels of all the other indicators were not significantly different between normal and test groups (*p* < 0.05).

### 3.3. HF detection using SPIO@SiO_2_–ICG–RGD by NIR imaging *in vivo*

**Fig. 4:**
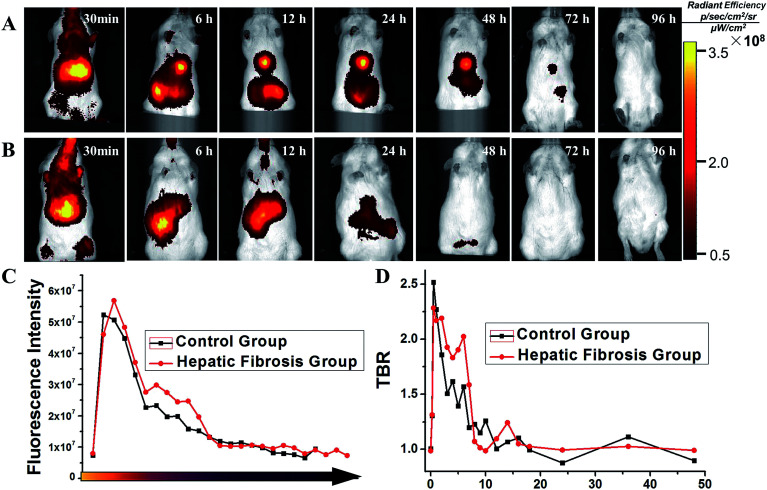
*In vivo* comparison of the biodistribution of SPIO@SiO_2_–ICG–RGD for different mouse models. (A) A model of hepatic fibrosis in mice. (B) Healthy mouse model (control). (C) Comparison of fluorescence intensity in the two groups. (D) Comparison of the target:background ratio (TBR) of the two groups.

Mice were divided into two groups: HF (*n* = 10) and control (*n* = 10). Then, 150 μL of SPIO@SiO_2_–ICG–RGD (0.2 mg mL^−1^) was injected intravenously into all mice. Continuous fluorescence imaging was undertaken up to 96 h using an *in vivo* optical imaging system (IVIS Spectrum) for both groups (Fig. 4A and B).

After the initial distribution period (<12 h), most fluorescence signals were emitted from abdominal areas. Then, the signal faded gradually after injection at different times in the HF (>72 h) and control (<24 h) groups. The images of SPIO@SiO_2_–ICG showed similar tendencies as the normal group with SPIO@SiO_2_–ICG–RGD (Fig. S2A and S2B†).

These results suggested that the probes were excreted through the liver and digestive system in control and HF groups; there was no substantial change in the metabolic pathway. However, the duration of the probe in the HF group was longer than that in the healthy group because HSCs with high expression of integrin α_v_β_3_ in mice in the HF group were bound to the RGD sequence in the SPIO@SiO_2_–ICG–RGD probe. Notably, differences between the HF and control groups were observed. The most significant contrast at the optical signal point of SPIO@SiO_2_–ICG–RGD appeared from 0.5 h to 3 h (*P* < 0.05), and the most significant contrast point appeared 2.5 h after injection (*P* = 0.002), with a value of (2.192 ± 0.0479) × 10^7^ (Fig. 4C); these results agreed with the TBR value in Fig. 4D, and the most significant contrast point value was 2.059 ± 0.0372.

### 3.4. HF detection using SPIO@SiO_2_–ICG–RGD by MRI *in vivo*

**Fig. 5:**
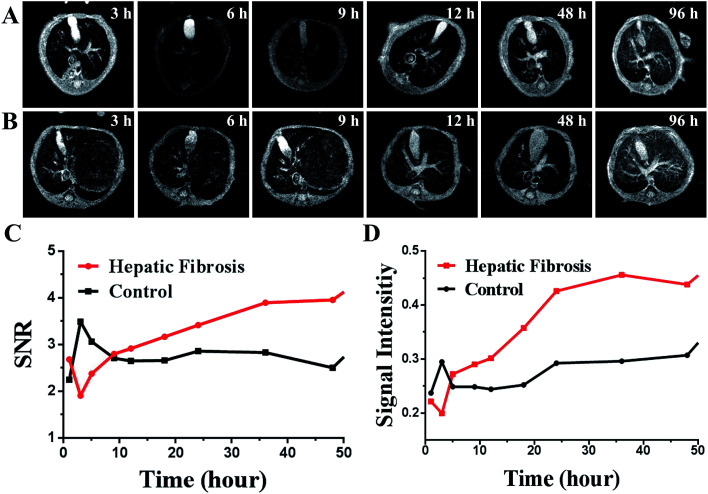
*In vivo* comparison of MRI of SPIO@SiO_2_–ICG–RGD for different mouse models. (A) A model of hepatic fibrosis in mice. (B) Healthy mouse model (control). (C, D) Comparison of MRI intensity in the two groups using the two methods.

MRI was done in the same mice used for NIR imaging. After intravenous injection with the SPIO@SiO_2_–ICG–RGD probe, mice underwent continuous MRI along with NIR imaging up to 96 h. The results shown in Fig. 5A and B indicate that the T2 MRI signal decreased continuously in the liver. The metabolism results were the same as those determined using NIR imaging. After gathering data, SI analyses were done (Fig. 5C) and double-checked (Fig. 5D). Differences between the SI_liver_-to-SD_background_ ratios (SNRs) for HF and control groups were compared (Fig. 5C). Moreover, compared with the normal group SPIO@SiO_2_–ICG–RGD, the data for SPIO@SiO_2_–ICG showed almost the same tendency as shown in Fig. S3A and B.† The visible contrast at the MRI signal point of SPIO@SiO_2_–ICG–RGD appeared from 1 h to 5 h (*P* < 0.05), and the most visible MRI signal peak contrast appeared 3 h after injection (*P* = 0.005), with a peak SNR of 3.494 ± 0.537. Differences in the liver-to-muscle ratios between the HF and control groups were assessed (Fig. 5D). The most visible MRI signal contrast point of SPIO@SiO_2_–ICG–RGD appeared 3 h after injection (*P* = 0.005), and the peak SI value was 0.3261 ± 0.0307. Using these two methods, similar trends were obtained, although the results revealed different values for the same point.

### 3.5. Histopathology

**Fig. 6:**
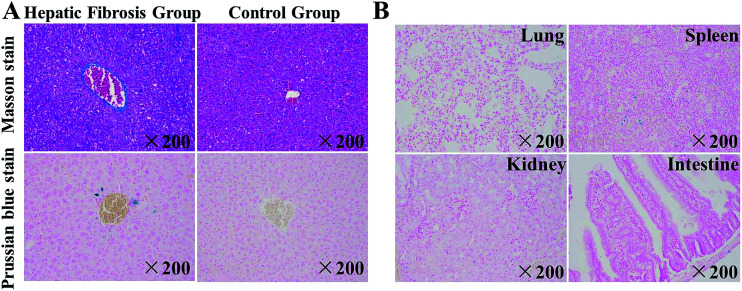
Histopathology of mice with hepatic fibrosis (HF) and healthy (control) mice. (A) Slides of liver tissue from HF mice and control mice after intravenous injection of SPIO@SiO_2_–ICG–RGD. A photograph of Masson stains and Prussian blue stains of liver tissue collected from the HF and control group of mice. (B) A photograph of Prussian blue staining from the lung, spleen, kidney and intestine of HF mice.

Liver tissue slices were prepared and subjected to staining (H&E, Masson, Prussian Blue). Fig. 6 shows the histopathology of spleen, lung, kidney, intestine, and liver tissues from mice in the HF group and that of liver tissue from mice in the control group.

Upon Masson staining, the distribution of collagen could be seen clearly, and HF tissue could be distinguished from normal tissue in the control group (normal liver) (Fig. 6A), whereas SPIO@SiO_2_–ICG–RGD induced a much higher level of staining of iron ions in the HF liver than in the normal liver (Fig. 6A). To detect the biodistribution of the probe, the main organs from mice in the HF group were stained with Prussian blue (Fig. 6B), which stained iron ions blue. Prussian blue staining of the lung, spleen, kidney, and intestine tissue from mice in the HF group indicated less staining of iron ions than that observed in the liver. These results suggested that SPIO@SiO_2_–ICG–RGD can be used as a HF imaging agent that can target normal liver tissue precisely.

## 4. Discussion

HF, as the only reversible process of chronic liver disease, remains a big diagnostic challenge. Noninvasive and effective methods have been developed to assess HF quantitatively.^13,22–25^ Using SPIO/ICG with an RGD-based targeting integrin α_v_β_3_ as a nanoprobe, we created SPIO@SiO_2_–ICG–RGD. The latter could be used to quantify and characterize the early stage of HF in mice induced by CCl_4_ in an accurate and noninvasive manner.

Here, we described the design and potential of SPIO@SiO_2_–ICG–RGD, which can be used for MRI–NIR imaging to accurately detect HF regions. To meet the challenge of developing a safe, convenient, and highly effective dual-mode imaging agent to diagnose HF, we chose SPIO and ICG as the photographic developers for T2 MRI and NIR imaging, respectively, which have been in clinical use for a long time.^30,31^ Compared with the conventional diagnostic method, NIR imaging and MRI could offer high sensitivity and spatial resolution for the early detection of HF with no radiation exposure, and could be a powerful tool for offering structural and functional imaging information. The high sensitivity of NIR imaging enabled detection of tiny amounts of ICG. In addition, because MRI can show sectional anatomic information clearly, it could detect changes in signal intensity gradually from the portal area to the liver capsule area, in accordance with histological analyses. However, ICG can be cleared rapidly by the liver,^37^ whereas SPIO may adhere to macrophages for a long time.^38^ To avoid these disadvantages of these two substances and to combine them together, we used a core–shell structure of SiO_2_ to “wrap” them. As revealed by XPS, there was a very small peak and less iron content on the surface of the composite, which showed that we had wrapped the Fe_3_O_4_ in the center successfully. Then, we used PEG, which increased the biocompatibility of the inorganic material (SiO_2_) in tissue.

For precise detection, we took advantage of the enhanced permeability and retention effect. When selecting the target, we found that the RGD-modified probe targeted HSCs that overexpressed integrin α_v_β_3_, and probe specificity was verified *in vitro* and *in vivo*.^39–41^ With specific binding of the RGD peptide to integrin α_v_β_3_, nanomaterials could be internalized within cells by clathrin-mediated endocytosis (also known as receptor-mediated endocytosis). Some studies have shown^14,36^ that the expression of integrin α_v_β_3_ increases significantly in the fibrotic liver, especially in advanced fibrosis, but not dramatically at the early stage. Meanwhile, the low sensitivity of detectors for imaging of early-stage HF by targeting integrin α_v_β_3_ restricts their use in clinics. Some studies have confirmed the target,^13,14,36^ but fewer studies have reported nanoprobe targeting of the other receptors used for molecular imaging of HF in animal models. Several studies have reported targeted therapy of HF,^42,43^ but we thought it was more important to diagnose it first.

After creation of SPIO@SiO_2_–ICG–RGD compound, toxicity assays confirmed its biocompatibility and safety *in vitro* and *in vivo*. Use of the targeting probe SPIO@SiO_2_–ICG–RGD for MRI and NIR imaging enabled qualitative and quantitative evaluation of HF in a timely manner (Fig. 4 and 5). The early HF induced in Kunming mice (the most widely applied animals in medical experiments in China) with CCl_4_ was confirmed by histology. The result clearly showed the synchronization of signal distribution. Fast clearance of compounds is a feature of the liver, but this feature was not seen in organs with lesions, mainly because the probe targeted overexpressed integrin α_v_β_3_ on HSCs. Non-use of the targeting probe SPIO@SiO_2_–ICG for MRI and NIR imaging revealed rapid clearance from fibrotic livers just like in normal mice (Fig. S2 and S3†). Hence, we observed greater accumulation of SPIO@SiO_2_–ICG–RGD in the fibrosis group, not only in MRI signals but also in NIR signals, than in the control group. This process repeats itself until clearance of the nanomaterial from the bloodstream is complete. Histopathology clearly demonstrated nanoprobe targeting of the liver just as we designed it, and SPIO@SiO_2_–ICG–RGD had nonspecific uptake into macrophages (Kupffer cells).

Thus, NIR imaging an MRI revealed that SPIO@SiO_2_–ICG–RGD can elicit accurate identification of fibrotic regions in the liver. This method provides valuable information for HF diagnosis. Nevertheless, a dual-mode probe capable of detecting HSCs in HF regions is required for further clinical application of this modality.

## 5. Conclusions

Core/shell nanoparticles of SPIO@SiO_2_–ICG–RGD were synthesized and utilized for *in vitro* and *in vivo* biomedical applications that were safe, convenient, and highly effective. Integrated Fe_3_O_4_ nanoparticles and ICG (which are both FDA-approved) coupled with RGD to target fibrotic cells and HSCs. The SPIO@SiO_2_–ICG–RGD probe targeted overexpressed integrin α_v_β_3_ on HSCs, and showed specific MRI and NIR imaging signals that demonstrated different clearance times from the liver. This dual-mode method could be used for HF detection. The present study provides empirical evidence to substantiate the promising potential of a SPIO@SiO_2_–ICG–RGD probe to diagnose liver disease.

## 6. Compliance with ethical standards

The authors declare that all experimental protocols were in compliance with ethical standards.

## 7. Ethical approval

This article does not contain studies with human participants undertaken by any of the authors. All animal experimental protocols were approved by the Institutional Animal Care and Ethics Committee of Capital Medical University, and all the methods were carried out in accordance with the relevant guidelines.

## Conflicts of interest

The authors declare that they have no conflict of interest.

## Supplementary Material

RA-008-C7RA10847H-s001
