# Supplementary material for: The diagnosis of hepatic fibrosis by magnetic resonance and near-infrared imaging using dual-modality nanoparticles
Source: RSC Adv. 2018 Feb 12;8(12):6699–708. doi: 10.1039/c7ra10847h (PMC9078292; doi:10.1039/c7ra10847h)
Supplement: RA-008-C7RA10847H-s001 [file RA-008-C7RA10847H-s001.pdf]

Electronic Supplementary Material (ESI) for RSC Advances.
This journal is © The Royal Society of Chemistry 2018

Hepatic Fibrosis Diagnose by Synergistic Magnetic Resonance and Near-Infrared Imaging with dual-modality nanoparticles

YunfangLi[1†], WentingShang[2†], XiaoyuanLiang[2], ChaotingZeng[2], MingmingLiu[1], Sudan Wang[1], HongjunLi[1*], JieTian[2*]

[1]Department of Radiology, Beijing YouAn Hospital, Capital Medical University, Beijing, 100069, China

[2]Key Laboratory of Molecular Imaging, Institute of Automation, Chinese Academy of Sciences, Beijing 100190, China

[†]The parallel first authors: Ms. Yunfang Li, Dr. Wenting Shang.

*The parallel corresponding authors: Prof. JieTian, Ph.D., Prof. Hongjun Li.

Professor Hongjun Li, M.D.

Director, Radiology department, Beijing YouAn Hospital, Capital Medical University

Address: 8 Xitoutiao, Youanmen, Fengtai district, Beijing, China

Zip code: 100069

Phone: +86 10 83997337; Fax: +86 10 63051128

E-mail: lihongjun64@126.com

Prof. Jie Tian, Ph.D., FIEEE, FSPIE, FIAMBE, FAIMBE

President, Chinese Society for Molecular Imaging

Professor and Director, Key Laboratory of Molecular Imaging, Institute of Automation, Chinese Academy of Sciences

Professor and Director, Beijing Key Laboratory of Molecular Imaging

Address: Zhongguancun East Road #95, HaidianDist, Beijing 100190, P. R. China

Phone: +86 10 82618465; Fax: +86 10 62527995

Email: jie.tian@ia.ac.cn

Supporting Information

Supplemental Figure1.

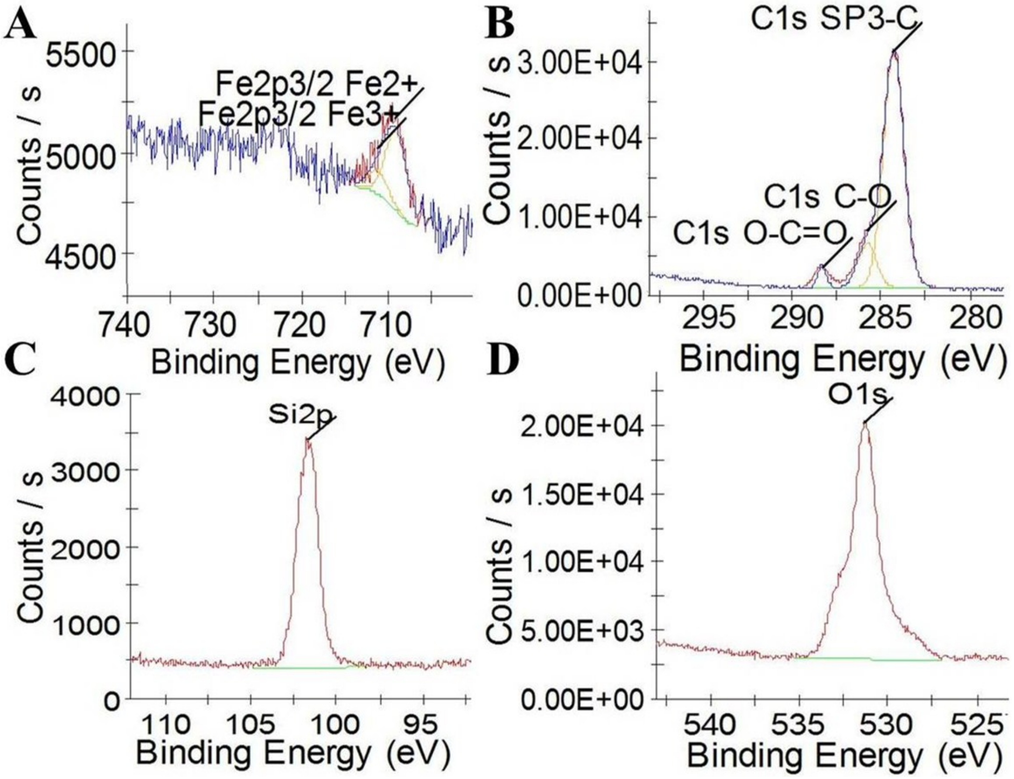

Supplemental Figure 1. The XPS high-resolution spectra of Fe2p(A), C1s(B), Si2p(C) and O1s(D) from the fractured surfaces of theSPIO@SiO2.

Supplemental Figure 2, 3

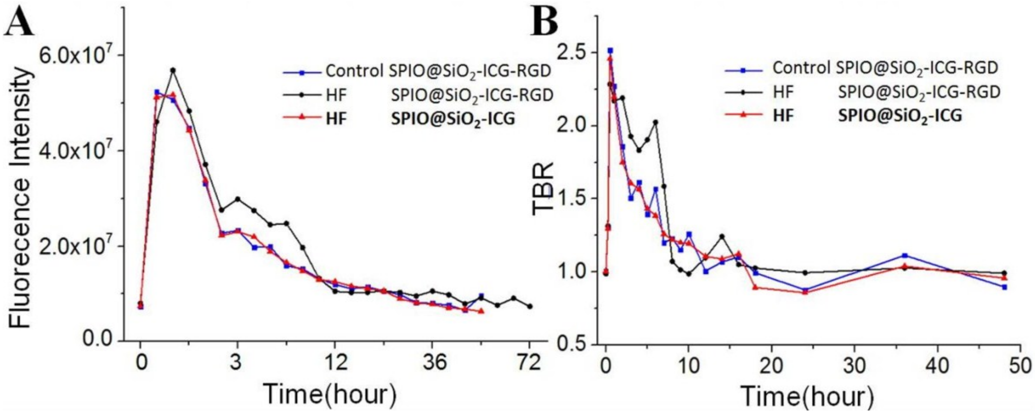

Supplemental Figure 2: *In vivo* biodistribution of the fluorescence intensity of SPIO@SiO2-ICG for hepatic fibrosis mouse models. (A) The fluorescence intensity profile of theSPIO@SiO2-ICG (red line). (B) The target to background ratio (TBR) profiles of the SPIO@SiO2-ICG (red line).

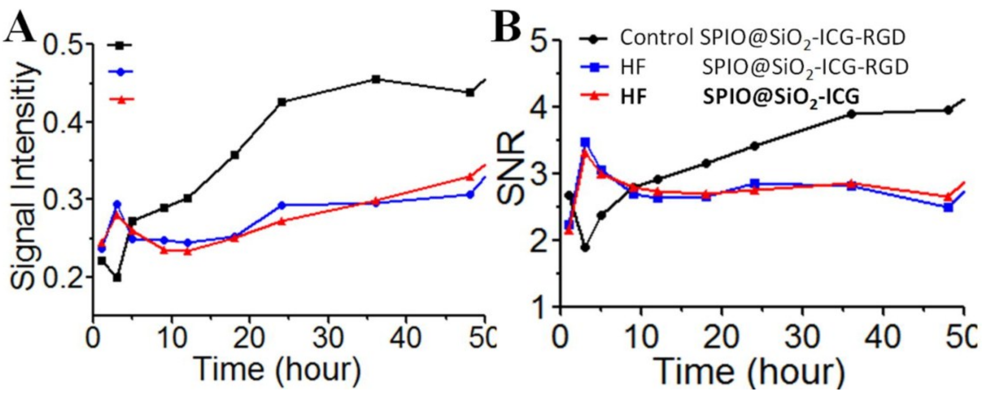

Supplemental Figure 3: *In vivo* biodistribution of MR intensity of SPIO@SiO2-ICG for hepatic fibrosis mouse models. (A, B) The MRI intensity profiles of the SPIO@SiO2-ICG (red line).

Supplemental Table 1: Serum Biochemical Analysis (meam±SD)

	Treatment group	Control group	*P* value
Aspartate aminotransferase (U/L)	74.33±128.75	81.70±71.06	0.072
Alanine aminotransferase (U/L)	50.97±44.24	15.27±11.48	0.006
Total bilirubin (μmol/L)	24.83±12.29	12.13±8.43	0.214
Direct bilirubin (μmol/L)	0.83±0.64	1.03±0.68	0.730
Total protein (g/L)	75.40±16.44	64.67±4.38	0.336
Albumin (g/L)	32.10±4.06	30.93±1.27	0.659
Globulin (g/L)	43.30±12.44	33.73±5.58	0.291
Gamma-glutamyltranspeptidase (U/L)	14.80±14.58	2.80±3.94	0.410
Alkaline phosphatase(ALP) (U/L)	36.60±3.99	63.43±53.30	0.475
Creatinine kinase (U/L)	9.12±1.50	9.91±0.71	0.238
Urea (mmol/L)	24.20±12.61	11.97±8.61	0.458
Uric acid (μmol/L)	51.93±22.05	78.50±35.25	0.331
Glucose (mmol/L)	13.50±5.53	14.09±2.24	0.873
Cholesterol (mmol/L)	2.32±0.06	2.08±0.49	0.444
Na (mmol/L)	137.37±8.35	142.23±2.63	0.390
Cl (mmol/L)	98.90±5.67	103.50±1.71	0.250

*$p<0.05$
